# An Analytics Platform to Evaluate Effective Engagement With Pediatric Mobile Health Apps: Design, Development, and Formative Evaluation

**DOI:** 10.2196/11447

**Published:** 2018-12-21

**Authors:** Quynh Pham, Gary Graham, Chitra Lalloo, Plinio P Morita, Emily Seto, Jennifer N Stinson, Joseph A Cafazzo

**Affiliations:** 1 Institute of Health Policy, Management and Evaluation Dalla Lana School of Public Health University of Toronto Toronto, ON Canada; 2 Centre for Global eHealth Innovation Techna Institute University Health Network Toronto, ON Canada; 3 Child Health Evaluative Sciences Research Institute The Hospital for Sick Children Toronto, ON Canada; 4 School of Public Health and Health Systems Faculty of Applied Health Sciences University of Waterloo Waterloo, ON Canada; 5 Department of Anesthesia and Pain Medicine The Hospital for Sick Children Toronto, ON Canada; 6 Lawrence S. Bloomberg Faculty of Nursing University of Toronto Toronto, ON Canada; 7 Institute of Biomaterials and Biomedical Engineering Faculty of Applied Science and Engineering University of Toronto Toronto, ON Canada

**Keywords:** analytics, engagement, log data, mobile health, mobile apps, chronic disease

## Abstract

**Background:**

Mobile health (mHealth) apps for pediatric chronic conditions are growing in availability and challenge investigators to conduct rigorous evaluations that keep pace with mHealth innovation. Traditional research methods are poorly suited to operationalize the agile, iterative trials required to evidence and optimize these digitally mediated interventions.

**Objective:**

We sought to contribute a resource to support the quantification, analysis, and visualization of analytic indicators of effective engagement with mHealth apps for chronic conditions.

**Methods:**

We applied user-centered design methods to design and develop an Analytics Platform to Evaluate Effective Engagement (APEEE) with consumer mHealth apps for chronic conditions and implemented the platform to analyze both retrospective and prospective data generated from a smartphone-based pain self-management app called *iCanCope* for young people with chronic pain.

**Results:**

Through APEEE, we were able to automate the process of defining, operationalizing, and evaluating effective engagement with *iCanCope*. Configuring the platform to integrate with the app was feasible and provided investigators with a resource to consolidate, analyze, and visualize engagement data generated by participants in real time. Preliminary efforts to evaluate APEEE showed that investigators perceived the platform to be an acceptable evaluative resource and were satisfied with its design, functionality, and performance. Investigators saw potential in APEEE to accelerate and augment evidence generation and expressed enthusiasm for adopting the platform to support their evaluative practice once fully implemented.

**Conclusions:**

Dynamic, real-time analytic platforms may provide investigators with a powerful means to characterize the breadth and depth of mHealth app engagement required to achieve intended health outcomes*.* Successful implementation of APEEE into evaluative practice may contribute to the realization of effective and evidence-based mHealth care.

## Introduction

### Background

The emergence of consumer mobile health (mHealth) apps for chronic disease self-management presents new opportunities and challenges for evidencing these novel interventions. Most consumer mHealth apps have not been evaluated for effectiveness on health outcomes [1]. This trend is particularly evident within the field of pediatrics, where recent reviews have revealed a paucity of evidence-based apps for young people across chronic conditions [2-4]. In spite of this, apps for pediatric chronic conditions are growing in availability [4] and challenge investigators to conduct rigorous evaluations that keep pace with mHealth innovations. Traditional research methods are poorly suited to operationalize the agile, iterative trials required to evidence and optimize these digitally mediated interventions [5,6].

In recent years, digital health researchers have called for novel methods to study engagement with digital health interventions [5]. They propose that engagement with an intervention is a precondition for effectiveness and warrants careful study to understand its relationship with the desired behavior change (eg, pain self-management) [7]. Yardley et al have furthered this focus on evaluating engagement by arguing that it may be more valuable to identify the mechanisms that underlie *effective engagement*, defined as “sufficient engagement with an intervention to achieve intended outcomes” [8]. They recommend the following 6 distinct methods to assess different aspects of effective engagement: (1) self-report interviews or observational sessions, (2) self-report questionnaires, (3) ecological momentary assessments, (4) system usage logs, (5) sensor data, and (6) psychophysiological measures.

On reviewing these methods, we noted that the majority can be delivered or collected from data generated by users directly engaging with a digital health intervention. These multilevel, temporally dense datasets may be sufficiently large to reliably model and experimentally test mediation of outcomes by engagement with particular intervention features and functionality, while statistically controlling for confounding moderator effects, such as baseline pain levels [9]. However, these data can also be complex [10,11], making it difficult to discern signal from noise [12,13]. Investigators may struggle to efficiently distill thousands of data points into meaningful insights that relate digitally mediated engagement with changes in health outcomes [14]. Realizing a method to cull through large mHealth app datasets and identify meaningful patterns of digitally mediated behavior change may promote a data-driven understanding of their impact on disease self-management.

### Objectives

Motivated by this understanding of the barriers to interpreting data from measures of effective engagement, we sought to contribute a resource to support the quantification, analysis, and visualization of analytic indicators of effective engagement with mHealth apps for chronic conditions. Specifically, we designed and developed an Analytics Platform to Evaluate Effective Engagement (APEEE) with consumer mHealth apps for chronic conditions and implemented the platform to analyze both retrospective and prospective data generated from a smartphone-based pain self-management app called *iCanCope* for young people with chronic pain [15]. Our intent was for APEEE to broadly enable investigators to query data being generated by users engaging with their mHealth apps in real time and specifically support the identification of mediating mechanisms that motivate effective engagement. This research assessed the feasibility of configuring APEEE for use in a pediatric research environment and its preliminary acceptability by mHealth investigators to inform evaluative practice. Specifically, (1) can the process of defining, operationalizing, and evaluating effective engagement with *iCanCope* be automated through APEEE? and (2) what are investigators’ perceptions regarding acceptability and satisfaction with APEEE?

This paper is organized as follows: first, we present the user-centered design (UCD) framework used to support the design and development of APEEE and review the features and functionality of the minimum viable product build of the platform. Second, we define the analytic indicators of effective engagement with *iCanCope* for inclusion in APEEE. Third, we review the technical and architectural considerations for modeling *iCanCope* engagement data and representing it on the platform. Finally, we describe the prototypical integration of APEEE with *iCanCope* to support a pilot randomized controlled trial (RCT) evidencing the intervention for young people with chronic pain.

## Methods

### The iCanCope App for Young People With Chronic Pain

Before initiating work on APEEE, we chose to identify a typical mHealth app to define our scope of work. Our rationale for establishing a single use case to guide the platform’s features and functionality was as follows: we wanted (1) a testing environment to experiment with various data integration and visualization methods, (2) to refine the platform’s computational capacity for modeling and managing dynamic data, (3) to validate data generated by the platform against data already being generated as part of an ongoing evaluation (eg, number of users, number of log-ins, and session duration), and (4) a direct route to implementation following development to trial our platform in evaluative practice. To meet these needs, we selected *iCanCope*, a smartphone-based pain self-management mHealth app tailored for adolescents and young adults aged 12 to 25 years with chronic pain [15].

The *iCanCope* project was conceived by the Improving Outcomes in Child Health through Technology (iOuch) research group, based out of the Hospital for Sick Children in Toronto, Canada [16]. The iOuch research group aims to improve the lives of children and adolescents through the use of innovative information and communication technologies. Research personnel includes a principal investigator, a research associate, 2 clinical research managers, 2 clinical research coordinators, 5 clinical research assistants, and a rotating roster of 5 to 7 research students and fellows. The group conducts research to conceptualize, design, and evidence digital health interventions such as *iCanCope* and outsources the development of the interventions to external research groups or software development studios. Moreover, 5 members of the research group are dedicated staff on the *iCanCope* project.

*iCanCope* was an appropriate match to inform our work because the app was already collecting data from participants in a pilot RCT to evaluate its preliminary efficacy on improving pain outcomes. Furthermore, our research group is the development partner on the *iCanCope* project, thereby ensuring ethical and direct access to both app data and the core research group evaluating the app. The main *iCanCope* features are (1) symptom tracking for pain intensity, pain interference, sleep, mood, energy, and physical activity in the form of daily *check* *-in* reports, (2) structured goal setting to improve pain and function, (3) an interactive toolbox of pain coping strategies, and (4) peer-based social support [15]. The app was developed natively for iOS and Android smartphone platforms. It was deployed in March 2017 for evaluation in a pilot RCT and had generated a significant amount of data before integration with APEEE in April 2018. We wish to note that although *iCanCope* features heavily in the conceptual narrative of APEEE, this research focuses on the platform as a proof-of-concept resource for pediatric mHealth app evaluation and as such does not constitute a study of *iCanCope* as an intervention for pediatric chronic pain.

### The User-Centered Design and Development of Analytics Platform to Evaluate Effective Engagement

The design and development of APEEE were guided by the UCD framework, which has been endorsed by the World Health Organization as a systematic approach to considering the needs of end users throughout all stages of the design life cycle [17]. As a design philosophy, the UCD framework endorses creating technology that users can, want, or need to use, rather than forcing users to change their behavior to accommodate the technology [18,19]. Starting with the *concept generation and ideation* processes in phase 1, user needs are identified to inform the intended goal of the digital health intervention. In phase 2, the *prototype design and system development* process is initiated, whereby identified user needs are translated into a set of functional requirements and design guidelines. Prototypes are created using these guidelines and refined through cycles of iterative design, often with real-time feedback elicited from end users. Phase 3 is the *evaluation* component of the process and ensures that the application can be implemented effectively in practice. Once these 3 phases are completed, the application is deployed to users. We initiated phase 1 of the UCD process in March 2018, progressed to phase 2 in May 2018, commenced phase 3 in June 2018, and advanced to a field study of APEEE in October 2018.

## Results

### Concept Generation and Ideation

We initiated phase 1 of the UCD framework by conducting a needs assessment session with 5 members of the iOuch research group to inform a baseline understanding of (1) their experience with the evaluation process, (2) their perception of the barriers and facilitators to evaluating the intervention, and (3) their definition of what constitutes effective engagement with the intervention. Investigators were prompted to speak about their specific evaluation questions, what measures were used to answer the evaluation questions, what data were required to operationalize those measures, and how that data had been collected. In parallel, we conducted a scoping review to identify and validate the terminology, definitions, and taxonomy of analytic indicators being used to measure effective engagement with mHealth apps for chronic conditions. Preliminary findings from the review informed the creation of a library of analytic indicators, which we referenced to define a shortlist of analytic indicators specific to *iCanCope*. Finally, we reviewed the existing *iCanCope* system architecture and data model to assess the feasibility of implementing the proposed shortlist of analytic indicators. We presented our recommendations to the iOuch research group for review and collaboratively finalized a list of 25 analytic indicators to represent on APEEE. Table 1 presents all analytic indicators, each expressed as a research question, and their corresponding definition.

### Prototype Design and System Development

To execute phase 2, we determined the design and development specifications required to represent each analytic indicator on APEEE. These specifications subsequently guided the selection of products to build out the platform as well as platform features and functionality. APEEE was developed using a collection of 3 open-source products: *Logstash*, *Elasticsearch*, and *Kibana* [20]. *Logstash* is a server-side data processing pipeline that ingests data from various sources simultaneously, executes different transformations, and exports the data to various targets. Given that data can be siloed across systems in different formats, Logstash supports data from logs, metrics, Web apps, data stores, and cloud computing services. As data travel from source to store, Logstash filters parse each event, identify named fields to build structure, and transform them to converge on a common format for analysis. *Elasticsearch* is a search engine based on the Lucene information retrieval software library. It provides a distributed, multitenant-capable, full-text search engine with a Web interface and schema-free JavaScript Object Notation documents. Elasticsearch allows users to perform and combine many types of searches, such as structured, unstructured, geographical, and metric. *Kibana* is an analytics and visualization plugin to Elasticsearch. Users can interface with Kibana to search, view, and interact with data stored in Elasticsearch indices. They can also perform advanced time-series analyses and visualize data in a range of charts, tables, and maps. Kibana facilitates the analysis of large volumes of data and also enables the creation of dynamic dashboards that display data queries in real time.

**Table 1:** Analytic indicators of effective engagement with *iCanCope*.

Analytic indicator		Definition
**Health status**		
	How are users doing on pain-related outcomes?	Raw and mean pain intensity, pain interference, sleep, mood, energy, and physical activity check-in scores generated over time
	Are users recording positive or negative check-in trends?	Number of positive and negative trends triggered
	Which pain-related outcome scores are users reporting the most?	Number of scores reported per check-in score response
	Which pain-related outcome scores are most users reporting?	Number of users reporting scores per check-in score response
**Check-** **ins**		
	How many check-ins are being completed daily?	Number of check-ins completed every day
	How many check-ins have been completed since study launch?	Number of check-ins completed in the last 90 days
	How many users have completed at least one check-in a day, every day, over the last 7 days?	Number of users with ≥1 check-in completed in a day, every day
	How many users have not completed a check-in for 7 consecutive days?	No check-ins logged for 7 consecutive days
	How long did it take for users to complete their first check-in?	Time between account creation and first check-in completed
	Which 10 users have completed the most check-ins?	Identity of user and number of check-ins completed
	How many check-ins were completed this week versus last week?	Number of check-ins completed this week and number of check-ins completed last week
**Goals**		
	Are users completing set goals?	Number of goals set and completed
	What types of goals are users setting the most?	Number of activity, sleep, energy, mood, and social goals set
	What types of goals are most users setting?	Number of users setting activity, sleep, energy, mood, and social goals
	How long did it take for users to complete their first goal?	Time between account creation and first goal created
**Community**		
	How many users have engaged with the community features?	Number of users who liked or made a post on the community feature
	What were the top 5 community questions with the most responses?	Content of community questions and number of responses
**Library**		
	What are the top 10 most popular library articles?	Content of library articles and number of reads
**History**		
	How many users accessed the history feature at least once?	Number of users who clicked on the history feature
	What symptoms are users reviewing in the history feature?	Contents and number of history pages clicked
**Other**		
	How many users have activated an *iCanCope* account?	Number of users registered on the study server
	How many users have logged any activity in the last 7 days?	Number of users who generated ≥1 event on the study server in the last 7 days
	How many users have logged any activity in the last 24 hours?	Number of users who generated ≥1 event on the study server in the last 24 hours
	Where in the world are users accessing the app?	Geolocation of user internet protocol addresses
	How far have users progressed in the study?	Numbers of days elapsed since account creation

Dashboards can be shared with a broader sample of users through URL weblinks, and analytic reports can be exported to comma-separated value (CSV) and PDF formats. To summarize, Logstash collects and parses log data, Elasticsearch indexes and stores the data, and Kibana visualizes the data to provide actionable insights. Together, these 3 open-source products are designed for use as an integrated solution, commonly referred to as the *Elastic Stack*.

This research primarily focused on configuring the Elastic Stack to conform with APEEE product specifications. Our decision to forego writing proprietary code in favor of adopting an open-source solution was dually motivated: (1) we wanted to leverage the Elastic Stack features, functionality, and documentation built by a community of 100,000 developers over 6 years [21], and (2) we are proponents of the open-source software development methodology [22]. Using Elastic Stack capabilities, we developed a prototype of APEEE that enabled investigators to (1) visualize a library of effective engagement analytic indicators extracted from *iCanCope* data; (2) build filters to segment the study population into cohorts for comparative analyses; (3) monitor the status of informed electronic consent, study progression, and fidelity of intended engagement by young people with chronic pain; (4) conduct basic statistical analyses on a dynamic engagement dataset; and (5) generate individual- and aggregate-level analytic insights in real time. The principal feature of APEEE is the *APEEE Dashboard*, which is an interface for investigators to view analytic indicator trends for immediate research-to-action application (eg, inform the need to modify an intervention feature because of poor engagement). To support platform functionality, we built a Personal Health Information Protection Act–compliant *APEEE Engine* to serve as the foundational information management and data infrastructure required to integrate and store engagement data and support data mining and export for advanced statistical analyses.

**Figure 1:**
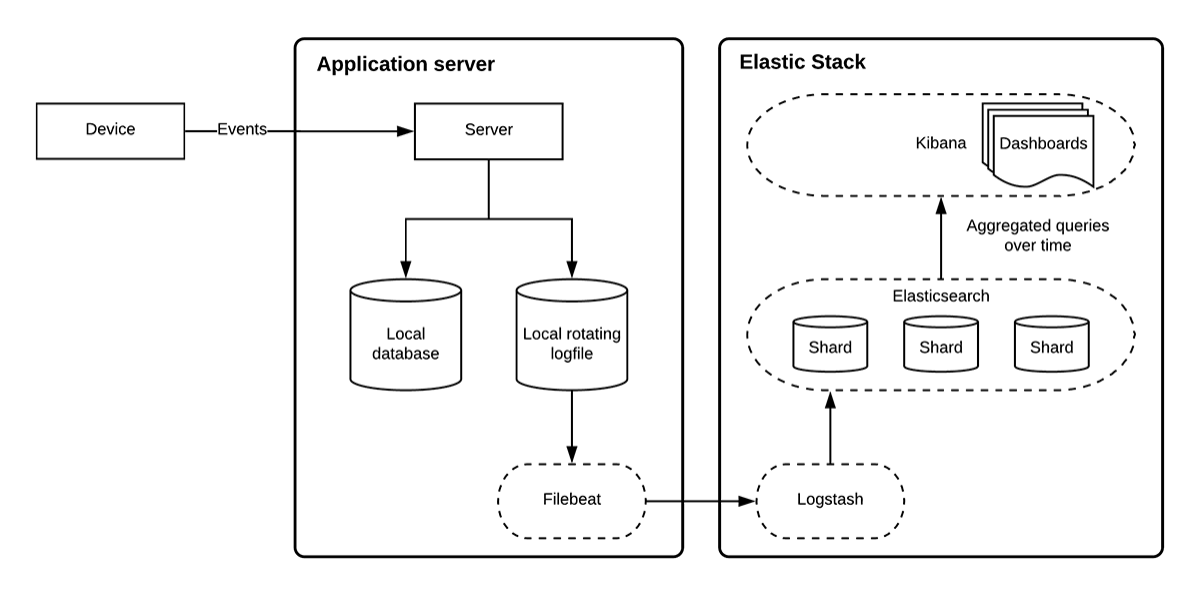
APEEE system architecture. APEEE: Analytics Platform to Evaluate Effective Engagement.

For APEEE to produce meaningful insights on engagement with *iCanCope*, we first needed to aggregate and store events generated from both client devices and the application server. To realize this work, we used an *event streaming* architecture, which lends itself well to analyzing temporally dense data. Furthermore, it provides us with the ability to see data as they were at any given point in time; this property is useful for conducting time-series analyses. At the most basic level, a unit of data is an *event* and contains 2 pieces of information: identification and payload. The former is used for aggregatory purposes and cohort tracking, and the latter is the actual event that occurred, along with any useful metadata. To illustrate the passage of our data from *iCanCope* to APEEE, we will use a simple event in which a participant signs into the app on their device. First, an event called *user_signed_in* is generated by the device, along with a time stamp and user identifier. This event is sent to our application server, which stores it to a local database and then emits it to a local log file. However, these data cannot be analyzed on the application server and must be forwarded to a destination that can effectively process it. There is a lightweight daemon (ie, a computer program that runs as a background process) called *Filebeat* running on the application server, which detects any new events in log files and forwards them over a private network to our instance of the Elastic Stack. On arrival at the Elastic Stack, the event is ingested via Logstash, and metadata are pulled out from the event to increase the overall amount of data we can later query and visualize. From there, the event is tagged as an analytic event and is sent to Elasticsearch for indexing and storage. Elasticsearch not only performs minor analysis on the incoming event but also provides it with durability and ease of lookup by duplicating it over replica shards. Once the data arrive at Elasticsearch, it can now be queried in Kibana, which supports our various visualizations and dashboards. Figure 1 relates this use case as a system architecture diagram of APEEE.

**Figure 2:**
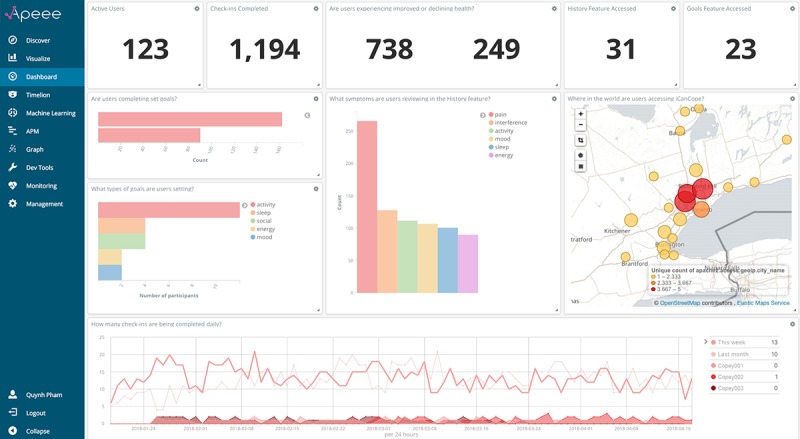
APEEE dashboard with a subset of analytic indicators of effective engagement with iCanCope. APEEE: Analytics Platform to Evaluate Effective Engagement.

Following platform configuration, we initiated the process of translating all analytic indicators into visualizations on the APEEE dashboard. This process involved selecting the appropriate graphic for each indicator (eg, line graph, pie chart, heat map, and data table), defining the appropriate data fields and parameters, and adjusting graphic assets (eg, axis values, table headers, and color schema) to represent the indicator as a dynamic visualization. We sought feedback from our internal team of human factors specialists and designers to ensure appropriate alignment between data and visualization. We also engaged in a rapid-cycle iterative prototyping process with the iOuch research group, where we sent over dashboard prototypes for review on a near-daily basis. This constant communication and collaboration with our end users allowed us to recalibrate our prototypes with emerging needs, which led to timely adjustments and improvements to the dashboard. Figure 2 presents the APEEE dashboard with a subset of finalized analytic indicators. To provide a comprehensive and instructive description of platform features and functionality, we have chosen to present 3 indicators in detail: (1) where in the world are users accessing *iCanCope*?, (2) what types of goals are users setting?, and (3) how many check-ins are being completed daily?

#### Where in the World Are Users Accessing iCanCope?

As a participant in the *iCanCope* pilot RCT, young people aged 12 to 25 years with chronic pain were instructed to download the app onto their personal device, create an account, and use the app as needed over the 8-week study period. Given that participants were free to engage with the app wherever they wanted, they subsequently generated an internet protocol (IP) address trail that we were able to access through analyzing their device log data. The opportunity to determine and map the physical location of a user’s IP address in real time was a novel challenge for our research group and a feature we wanted to trial in APEEE. We used Logstash’s *GeoIP* database to convert IP addresses into latitude and longitude coordinate pairs, which were then stored in Elasticsearch as *geo_point* fields and converted into *geohash* strings. Kibana was then used to read the *geohash* strings and draw them as points on a global map.

**Figure 3:**
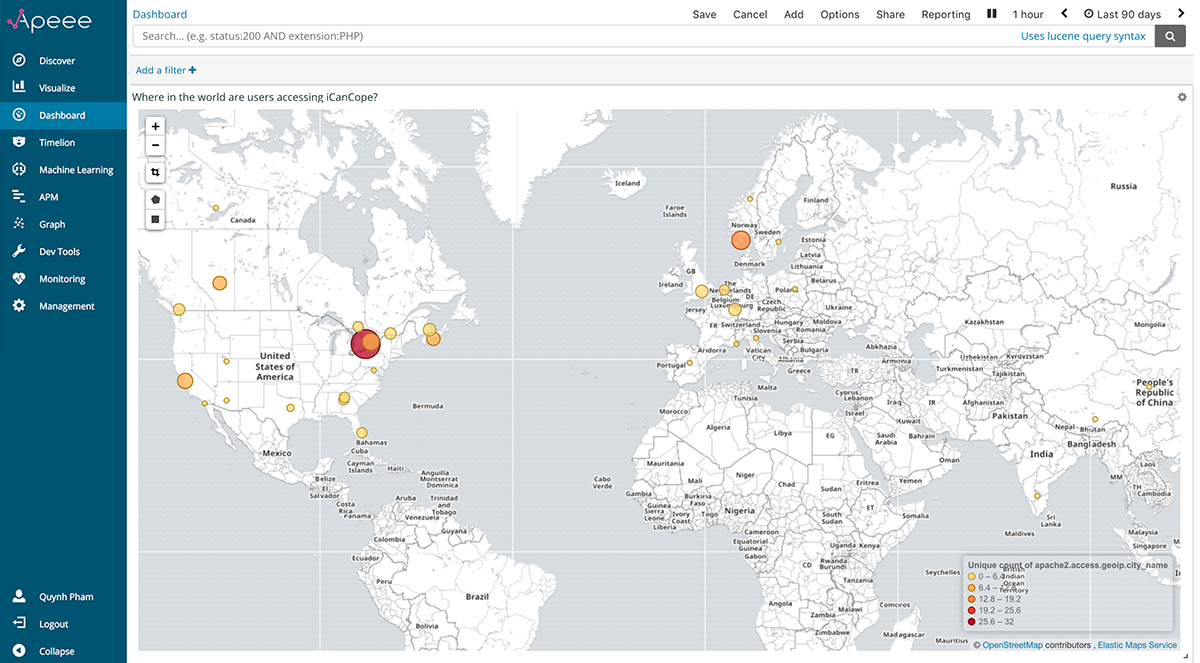
The analytic indicator for “where in the world are users accessing iCanCope?,” visualized through APEEE as a choropleth map covering 5 continents. APEEE: Analytics Platform to Evaluate Effective Engagement.

**Figure 4:**
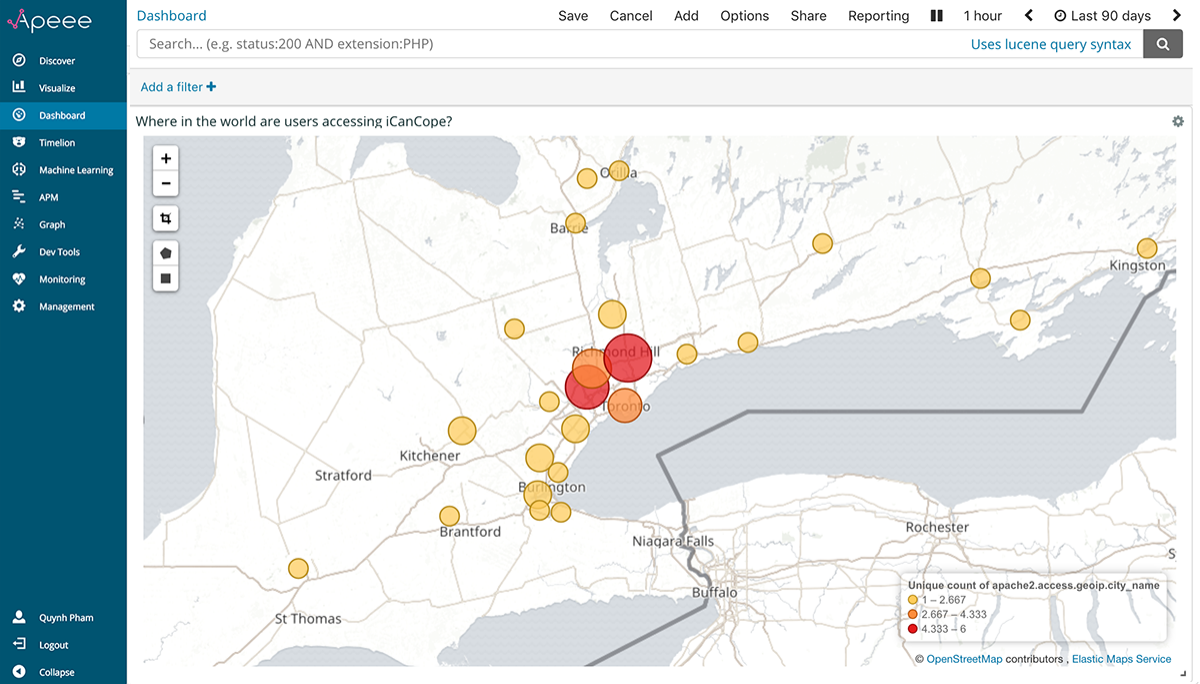
The analytic indicator for “where in the world are users accessing iCanCope?,” visualized through APEEE as a choropleth map covering the greater Toronto area in Ontario, Canada. APEEE: Analytics Platform to Evaluate Effective Engagement.

Figure 3 presents this analytic indicator, visualized through APEEE as a choropleth map covering 5 continents, with scaled circle markers representing the number of unique IP addresses logged by participants accessing *iCanCope*. Higher intensity colored circles indicate a greater concentration of addresses in a particular region. Investigators can click on a specific region of interest to view a narrowed spread of addresses; Figure 4 presents the view generated from repeatedly clicking on the large red circle in Figure 3 to plot participants across the greater Toronto area in Ontario, Canada. This geographical insight allows the iOuch research group to (1) validate whether participants are accessing the intervention in the community, (2) measure the geographical scale and spread of intervention access, and (3) observe shifts in access and engagement patterns over time.

#### What Types of Goals Are Users Setting?

A core component of *iCanCope* is the Goals feature, where young people are guided in setting structured goals aimed at improving their pain and function. Goals can be categorized across 5 domains: sleep, mood, energy, physical activity, and social activity. Given the hypothesized importance of this feature in promoting positive behavior change, we wanted to explore what types of goals participants were setting to understand what aspects of their behavior were amenable to improvement. We used Elasticsearch’s aggregations framework to build a summary of all goals set by participants throughout the trial. An aggregation can be considered a unit of work that builds analytic information over a set of data. For this work, we specifically applied 6 *bucketing* aggregations to our full set of study data: 1 parent aggregation for all goals completed and 5 nested subaggregations for each goal domain. When bucketing aggregations are executed in Elasticsearch, criteria for each bucket are evaluated against all data in a given set; if a criterion matches, the data *fall into* the relevant bucket.

**Figure 5:**
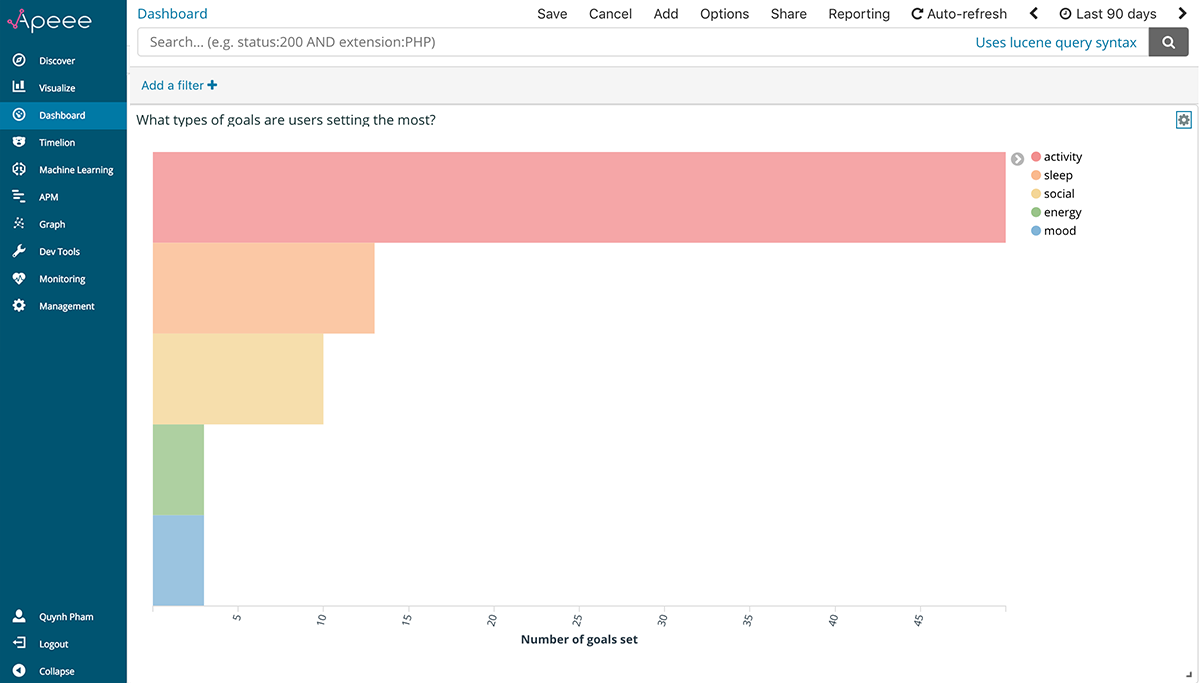
The analytic indicator for “what types of goals are users setting the most?,” visualized through APEEE as a horizontal bar chart. APEEE: Analytics Platform to Evaluate Effective Engagement.

**Figure 6:**
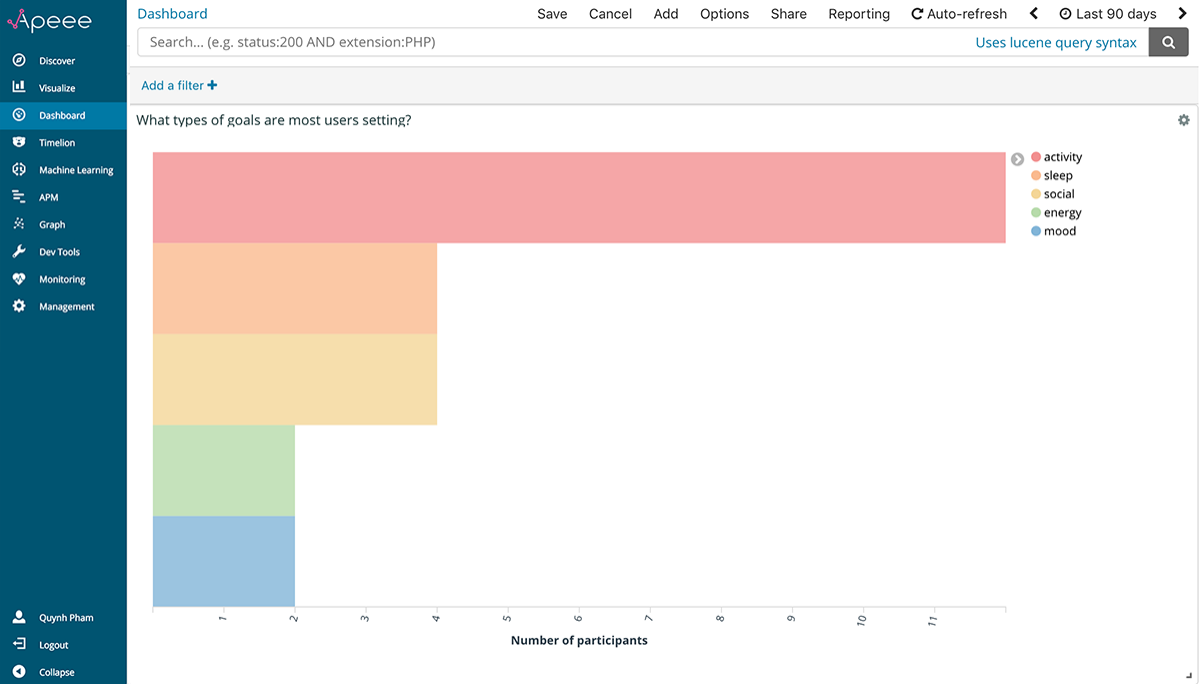
The analytic indicator for “what types of goals are most users setting?,” visualized through APEEE as a horizontal bar chart. APEEE: Analytics Platform to Evaluate Effective Engagement.

Figure 5 presents this analytic indicator, visualized through APEEE as a horizontal bar chart, with the y-axis representing goal domains and the x-axis representing the number of goals set. A color-coded legend on the right side of the chart identifies the domain for each bar. This graph indicates that participants are setting more physical activity goals than other goal types as a group. However, to ensure that findings were not being skewed by a small number of users setting a large number of physical activity goals, we accessed Kibana settings and changed the x-axis to represent the number of participants setting goals for each domain. This axis change and the consequent graph generated (Figure 6) were implemented in under a minute and allowed us to instantly corroborate both user-level and event-level insights. With this knowledge, investigators might, for example, design more physical activity goals for participants to browse and set.

#### How Many Check-Ins Are Being Completed Daily?

As part of the *iCanCope* trial, participants were asked to adhere to a symptom tracking protocol, aimed at helping them to recognize and understand patterns in their pain and functioning, and better communicate their symptoms with health care providers. This protocol was delivered through the check-in feature of the app, which prompted participants to complete a check-in a day for 56 consecutive days (ie, the duration of the trial). Participants tracked symptoms across 6 domains: pain intensity, pain interference, sleep, mood, energy, and physical activity. At the time of app integration with APEEE, more than 50 participants were enrolled in the trial and had collectively logged more than 3000 data points across all symptom domains. This temporally dense ecological momentary assessment dataset enabled us to develop time-series data parsing, analysis, and visualization functionality into APEEE. To realize this feature, we implemented Elastic’s aggregations framework and applied 2 bucketing aggregations to our data: (1) aggregating all check-ins logged by participants since study launch and (2) aggregating the number of daily check-ins over time. We then applied Kibana’s *time series visual builder* filter over our data to visualize insights.

**Figure 7:**
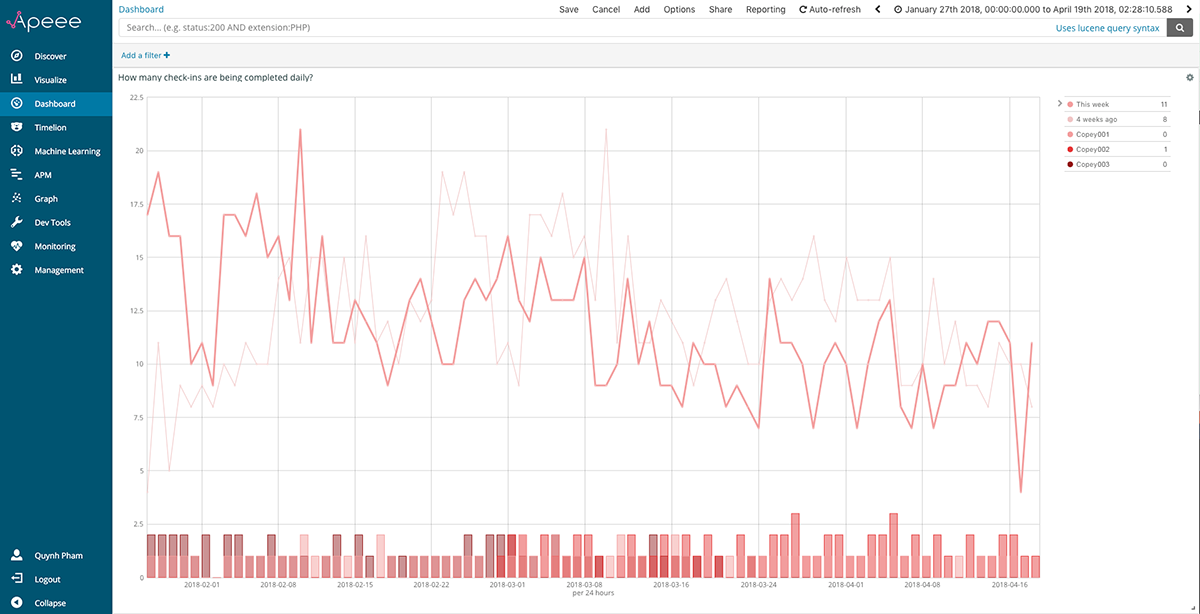
The analytic indicator for “how many check-ins are being completed daily?,” visualized through APEEE as a histogram with 3 layered graphs. APEEE: Analytics Platform to Evaluate Effective Engagement.

Figure 7 presents this analytic indicator, visualized through APEEE as a histogram with 3 layered graphs. The y-axis represents the total number of check-ins completed, and the x-axis represents time; the selected time range is 90 days. The first bolded line graph denotes the total number of check-ins completed per day. The second thin line graph also denotes the total number of check-ins completed per day but offset by 4 weeks. The third vertical bar chart with 3 superimposed bars denotes the 3 participants who have logged the most check-ins over the selected time range; participants were identified through a real-time count of check-ins conducted on the back end of the platform. Participants’ usernames are presented in the legend but have been changed to maintain confidentiality. This layering of analytic insights might allow investigators to understand, for example, if there is a widening gap between daily check-in counts this week versus 4 weeks ago or the extent of check-in contribution from highly engaged participants.

In summary, these 3 functional use cases serve to illustrate the potential for APEEE to support investigators in their evaluative practice. We aim for the real-time analysis and visualization of analytic indicators through APEEE to provide investigators with timely and meaningful insights, which can then be further investigated outside of the platform using qualitative measures of effective engagement [8].

### Evaluation

To operationalize phase 3 of the UCD framework, we conducted (1) 2 iterative cycles of evaluation on APEEE; the first with 2 members of the iOuch research group and the second with 7 members and (2) a between-cycle round of design and development. The first evaluation cycle was intended to assess the usability and acceptability of the platform and identify critical design and development requirements to be addressed and validated in the second evaluation cycle.

We first conducted a 1-day on-site observation session with 2 members of the iOuch research group to evaluate their initial use of APEEE. We were unable to provide investigators with direct access to the platform from their own devices because of the ongoing work at the time on the APEEE Engine. Instead, 1 member of our research group installed an instance of the platform onto a laptop, traveled to the evaluation site, connected to the APEEE Engine through a virtual private network, and launched the platform for use by the research group. Investigators were first provided with an overview of platform features and functionality and then presented the APEEE dashboard with visualizations for all *iCanCope* analytic indicators. They were then encouraged to explore each visualization and *think aloud* about the data representation and design specifications. Investigators were also asked to work independently through the following tasks while simultaneously verbalizing any difficulties encountered: (1) filtering visualizations by time range, (2) expanding a visualization to see more granular data points, (3) rearranging visualizations on the dashboard, (4) sorting numerical and string data table visualizations, and (5) exporting data table visualizations for download as CSV files. Field notes were taken during the session to record any technical difficulties encountered, ease of use, and learnings as well as nonverbal behaviors related to acceptability. Suggestions made by investigators on platform features or functionality that were not identified during the *concept generation and ideation* phase were considered for incorporation into the platform.

Overall, investigators found APEEE to be an acceptable resource to support their evaluation of *iCanCope*. They were able to independently work through all tasks with minimal guidance and sought clarification out of curiosity rather than necessity. There were no software bugs detected or system error codes returned during the session. The platform was explored with relative ease; however, some minor difficulties encountered included (1) inexperience with platform navigation, resulting in redundant actions to perform a task; (2) confusion regarding variable names, which retained server nomenclature and were sometimes difficult to interpret (eg, *clientCreated* to represent “participant”); and (3) unfamiliarity with performing Boolean searches using the Lucene query syntax, which is the default search syntax in APEEE. To alleviate these issues, we encouraged investigators to practice navigating the platform interface and repeating tasks until they felt intuitive and also provided them with a copy of the *iCanCope* data dictionary and a link to the Lucene query syntax as reference documentation. Investigators were satisfied with these additional resources and were able to complete tasks independent of them by session end. They saw potential in APEEE to accelerate and augment evidence generation both during and after trial conduct and expressed enthusiasm for adopting the platform as part of their evaluative practice. Suggestions to improve platform features and functionality included (1) partitioning the main dashboard into multiple subdashboards, each relating to a different feature in *iCanCope*; (2) supporting visualizations of events over *relative* time (eg, number of users who completed a check-in as a function of time elapsed in the study); (3) computing advanced predictive statistical analyses (eg, linear regressions); and (4) enabling remote access to APEEE. These requirements were feasible in scope and served as motivation to further develop the platform before full deployment.

#### Partition Dashboard and Enable Remote Access

Following this observation session, we initiated a new round of iterative design and development informed by the identified requirements. We were able to apply Kibana functionality and partition the main dashboard into multiple subdashboards. We also leveraged this functionality to build out custom dashboards for 5 members of the iOuch research group. Screenshots of these dashboards were presented to their intended users for review and found to be more useful than a single generic dashboard. To enable access to these dashboards for further testing and also address the remote access requirement, we activated Elasticsearch’s *Security* module and configured the *Authorization* functionality. Authorization in APEEE is the process of determining whether the user behind an incoming request is allowed to execute it. APEEE manages the privileges of users through *roles*. A role has a unique name and identifies a set of permissions that translate to privileges on secured resource. For example, we defined the *iCanCope research analyst* role on APEEE to have *read* privileges on all documents that match the query *action: checkin_completed*. This role is limited to only viewing check-in data, as opposed to the *iCanCope research coordinator* role that has to *manage* privileges on the *iCanCope* cluster and can view, edit, and delete all documents.

Once we had defined a series of roles that aligned with the management structure of the iOuch research group, we assigned them to the 5 users for whom custom dashboards had been built out. We added username and password functionality for all user accounts and then sent each user a Secure Sockets Layer encrypted link to their custom APEEE dashboard for testing. All 5 users were able to remotely access APEEE, log into the platform, and view their custom dashboard. We asked users to remotely access APEEE 3 more times throughout the day and then concluded testing by changing their passwords to withdraw access to the platform.

#### Visualize Engagement Outcomes Over Relative Time

The requirement for APEEE to support visualizations of events over relative time was of high priority for us to build out. We recognized the significant value that this functionality would add to APEEE, specifically in a research context where events are typically analyzed as a function of time elapsed in a study. To address this requirement, we sought to modify our existing analytic indicator for “how many check-ins are being completed daily” to have the x-axis represent time elapsed in the study. Visualizing check-in completion over time elapsed in the study supports determining effective engagement with *iCanCope* because the behavior of checking into the app and reporting symptoms is theorized to mediate improved pain-related outcomes [15]. We initiated work on this visualization by reviewing the *iCanCope* data model to determine the exact event that signified a user enrolling into the pilot RCT. Following discussions with the iOuch research group to clarify the enrollment protocol and validate the order of operations, we selected the first time a user logged into *iCanCope* as the *genesis event* from which to start recording time elapsed in the study. We then modified the *iCanCope* data model to generate a *daysSinceGenesis* metadata tag on every event logged, thereby enabling events to be positioned along the study timeline. Once this new metadata tag was deployed and tested, we sought to visualize the number of users who completed a check-in over time elapsed in the study. To create this visualization, we implemented Elasticsearch’s aggregations framework and applied 3 bucketing aggregations to our data: (1) aggregating all check-ins logged by participants since study launch, (2) aggregating the number of daily check-ins over time elapsed in the study (eg, days 0-56), and (3) aggregating check-ins by study allocation, which was a metadata tag that was already exposed on all *iCanCope* log data. We then applied Kibana’s *line graph* filter over our data to visualize insights.

**Figure 8:**
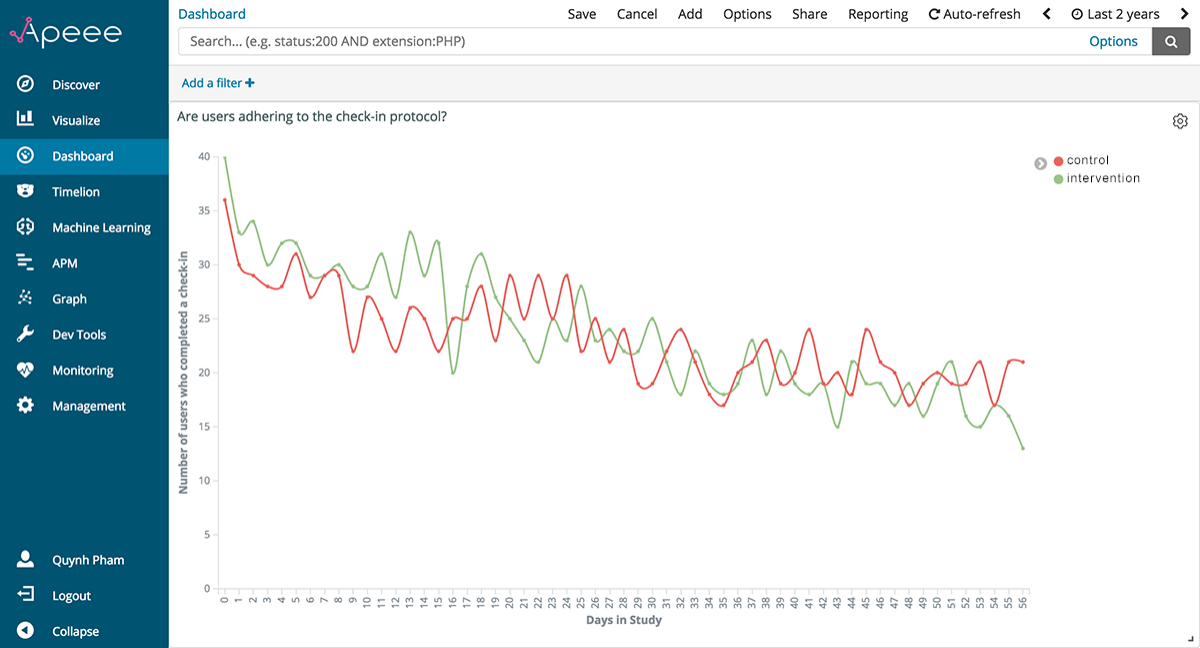
The analytic indicator for “are users adhering to the check-in protocol?,” visualized through APEEE as a line graph. APEEE: Analytics Platform to Evaluate Effective Engagement.

Figure 8 presents the visualization for the number of users who completed a check-in over time elapsed in the study, visualized through APEEE as a line graph, with the y-axis representing the number of users who completed a check-in and the x-axis representing the number of days elapsed in the study; the selected time range is 2 years. A color-coded legend on the right side of the chart identifies the study allocation for each line. With this relative time functionality, APEEE can support investigators to (1) monitor engagement outcomes in real time and (2) assess emerging outcome patterns and shifts across study groups over time.

#### Visualize Clinical Outcomes Over Relative Time

Equipped with the ability to create relative time visualizations, we endeavored to trial a final visualization before concluding our development cycle: a line graph of pain-related outcome scores reported by users over time elapsed in the study. An advantage to *iCanCope* was the in-app collection of clinical outcomes through the check-in feature. These data were stored on our servers as Fast Healthcare Interoperability Resources (FHIR), which is a data format that cannot be visualized on APEEE. We resolved this interoperability issue by transforming the FHIR data into log data through parsing out the outcome scores, injecting related user-level metadata, and then ingesting these data into Elasticsearch using Logstash. Once ingested, we applied the same Elasticsearch framework and aggregations for querying check-in data over time elapsed in the study.

**Figure 9:**
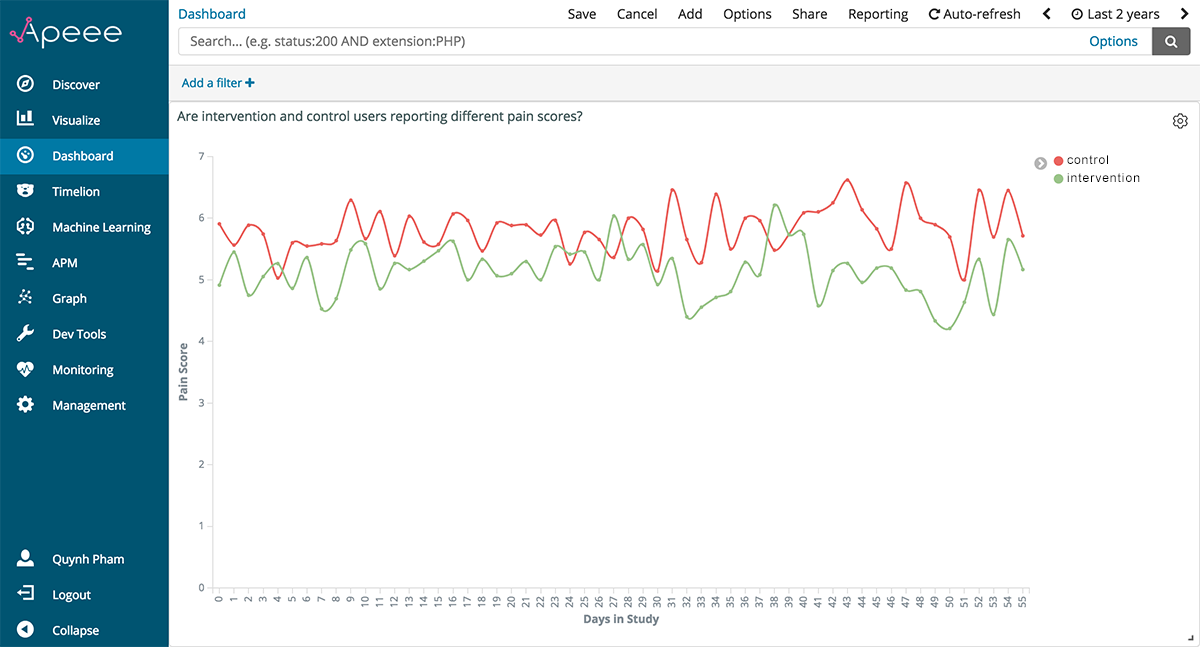
The analytic indicator for “Are intervention and control users reporting different pain scores?,” visualized through APEEE as a line graph. APEEE: Analytics Platform to Evaluate Effective Engagement.

Figure 9 presents the visualization for pain scores reported by users over time elapsed in the study, visualized through APEEE as a line graph, with the y-axis representing pain scores and the x-axis representing the number of days elapsed in the study; the selected time range is 2 years. A color-coded legend on the right side of the chart identifies the study allocation for each line. The ability to monitor real-time changes to clinical outcomes over the course of a study may encourage investigators to adopt innovative methodologies in their mHealth evaluations [23,24].

A live demo of the updated APEEE platform, including both relative time visualizations, was presented to 7 members of the iOuch research group during a weekly laboratory meeting at the evaluation site. The visualizations were well received, and the represented data were perceived to be significantly more useful when graphed over relative time. Investigators were particularly surprised by the sustained pattern of adherence to the check-in protocol by users in the control group and engaged in a spirited discussion of the potential motivations for this behavior. Overall, investigators found the updated build of APEEE to better meet their evaluative needs. A collective decision was made to proceed with a full deployment of the platform to the iOuch research group as part of a field study in October 2018.

## Discussion

### Principal Findings

At a time of rapid advancement in the mHealth field, evaluations of pediatric mHealth apps for chronic conditions must keep pace to increase the volume of evidenced apps made available to young people [25]. A shift toward adopting data-driven research methods would mark a significant development for the field, which has historically been “data-rich but evidence-poor” [26,27]. We posit that the adaptation of pediatric mHealth apps at the right time and under the right circumstances can accelerate evaluative practice and improve health outcomes. In this paper, we have shown that the process of defining, operationalizing, and evaluating effective engagement with *iCanCope* can be automated through APEEE. To our knowledge, APEEE is the first application of the Elastic Stack in a digital health context to support mHealth evidence generation. Configuring the platform to integrate with the app was feasible and provided investigators with a resource to consolidate, analyze, and visualize engagement data generated by participants in real time. Preliminary efforts to evaluate APEEE showed that investigators perceived the platform to be an acceptable evaluative resource and were satisfied with its design, functionality, and performance. Furthermore, they expressed enthusiasm for adopting the platform to support their evaluative practice once fully implemented. Future research is required to formally evaluate the impact of the platform on evaluative practice and mHealth app effectiveness.

### Limitations

Some methodological and functional limitations of our research warrant discussion. First, having a small number of members from a single research group participate in our evaluation was a major limitation and may have introduced bias, given the likelihood of shared perspectives. Second, our decision to build APEEE using the Elastic Stack exposes the platform to open-source updates made by the community of Elastic developers. This effectively means that changes may be pushed to APEEE’s features and functionality and implemented with little warning. We perceive this risk to be minimal and acceptable for the following reasons: (1) since initiating work on APEEE, all updates to the Elastic Stack have added value to the platform (eg, faster Elasticsearch queries, streamlined Kibana visualization builder) at no cost to our project, and (2) we are able to overwrite undesirable changes through branching the Elastic Stack source code and writing a version of the code for APEEE. Third, we were not able to address the suggestion for APEEE to compute advanced predictive statistical analyses in time for validation during our second evaluation cycle. We have since been able to graphically represent a series of probability distributions (eg, box plots and scatter plots) and interval estimations (eg, CIs and error bars) on APEEE using the Vega visualization grammar, which is a declarative language for building interactive graphs [28,29]. We will continue these preliminary explorations into VEGA-enabled predictive modeling and aim to validate this functionality in the field study of APEEE. Finally, although we were able to connect *iCanCope* to APEEE with relative ease, this process may not be indicative of the work effort required to connect a third-party mHealth app that we did not develop. APEEE benefits from the extensive Elastic Stack documentation and community resources (eg, blogs, YouTube videos, and forums) that detail the technical work effort required for connection [30]. However, the service design considerations for this connection are consequential [31] and may include (1) obtaining research ethics approval, (2) drafting data sharing agreements, and (3) reaching a shared understanding of what constitutes effective engagement and how to interpret analytic insights.

### Conclusion

Dynamic, real-time analytic dashboards such as the one discussed in this paper provide investigators with a powerful means to characterize the breadth and depth of mHealth app engagement required to achieve intended health outcomes. Through APEEE, participant engagement with *iCanCope* can be modeled with pain-related outcomes data to provide data-driven and actionable feedback. For example, daily check-in frequency can be analyzed against pain severity to inform a contextualized interpretation of app effectiveness. Using this information, the evaluative approach to evidencing *iCanCope* and its modular features can be optimized. Indeed, APEEE may enable the identification of digital biomarkers across chronic conditions for use in developing predictive engagement algorithms to tailor the content and timing of mHealth intervention delivery. In this way, the platform may contribute to the realization of effective and evidence-based mHealth care.

## Abbreviations

APEEE: Analytics Platform to Evaluate Effective Engagement
CSV: comma-separated value
FHIR: Fast Healthcare Interoperability Resources
iOuch: Improving Outcomes in Child Health through Technology
IP: internet protocol
mHealth: mobile health
RCT: randomized controlled trial
UCD: user-centered design
